# Understanding the Influence of Gypsum upon a Hybrid Flame Retardant Coating on Expanded Polystyrene Beads

**DOI:** 10.3390/polym14173570

**Published:** 2022-08-30

**Authors:** Sangram P. Bhoite, Jonghyuck Kim, Wan Jo, Pravin H. Bhoite, Sawanta S. Mali, Kyu-Hwan Park, Chang Kook Hong

**Affiliations:** 1 School of Chemical Engineering, Chonnam National University, Gwangju 61186, Korea; 2 HDC HYUNDAI EP R & D Center, Yongin-si 16889, Korea; 3 Department of Chemistry, Kisan Veer Mahavidyalaya, Wai 412803, Maharashtra, India

**Keywords:** hybrid flame retardant materials, influence of gypsum, minimum total heat release

## Abstract

A low-cost and effective flame retarding expanded polystyrene (EPS) foam was prepared herein by using a hybrid flame retardant (HFR) system, and the influence of gypsum was studied. The surface morphology and flame retardant properties of the synthesized flame retardant EPS were characterized using scanning electron microscopy (SEM) and cone calorimetry testing (CCT). The SEM micrographs revealed the uniform coating of the gypsum-based HFR on the EPS microspheres. The CCT and thermal conductivity study demonstrated that the incorporation of gypsum greatly decreases the peak heat release rate (PHRR) and total heat release (THR) of the flame retarding EPS samples with acceptable thermal insulation performance. The EPS/HFR with a uniform coating and the optimum amount of gypsum provides excellent flame retardant performance, with a THR of 8 MJ/m^2^, a PHRR of 53.1 kW/m^2^, and a fire growth rate (FIGRA) of 1682.95 W/m^2^s. However, an excessive amount of gypsum weakens the flame retardant performance. The CCT results demonstrate that a moderate gypsum content in the EPS/HFR sample provides appropriate flame retarding properties to meet the fire safety standards.

## 1. Introduction

Fire safety via the use of insulating materials is of prime priority in secure building construction. In the last two decades, expanded polystyrene foam (EPS) has become one of the main products in the insulation market due to its moisture resistance, good chemical resistance, and excellent thermal insulation [1,2,3,4]. Nevertheless, the highly flammable nature of EPS foam limits its application in the construction industry [5,6]. In recent years, many serious fire tragedies have resulted from the poor flame retardation properties of EPS foam. This represents a serious threat to civilian lives [7,8]. Therefore, it is an immense challenge for industries and researchers to boost the flame resisting properties of EPS foam. Nowadays, researchers focus on the incorporation of various flame retarding materials onto the EPS foam in order to enhance its fire retarding performance, with halogen-free flame retardants now being widely used in the academic and industrial sectors [9].

Among the various flame-retardant additives, intumescent flame retardants (IFRs) are widely used due to their environmental friendliness, low smoke production, and nontoxic properties [10,11]. It is well known that multiple phenomena occur during the combustion of polymeric materials. Thus, during the combustion of EPS foam, the IFR can generate a homogeneous protective char layer that both acts as a barrier to oxygen and heat, and suppresses smoke production, thereby enhancing the flame-retardant capability of the underlying materials [12,13,14,15]. In previous work, we prepared a flame-retardant expanded polystyrene foam, and found that, during combustion, the IFR material produced an expanded char layer which acted as an insulating barrier to inhibit heat transfer [16]. Therefore, the formation of an effective and continuous protective char layer is regarded as important for boosting flame retardancy. However, traditional IFR additives are less efficient than halogen-based flame retardants and require significant loadings in order to meet the desirable flammability standards [17]. To overcome this issue, studies suggest that the combination of multiple flame-retardant elements to achieve a synergistic effect would be the best choice [18]. Nevertheless, halogenated flame retardants are still the most efficient flame-retardant materials, and while they may cause environmental issues in some situations, there remains no promising alternative. For example, the bromine-containing molecule decabromodiphenyl ethane (DBDPE) greatly facilitates the gas phase activity of this flame retardant. Several studies suggested that DBDPE does not release the toxic and carcinogenic polybrominated dibenzo-p-dioxin (PBDD) and polybrominated dibenzofuran (PBDF) gases during combustion due to the absence of ether linkages [19,20,21,22,23,24].

Meanwhile, numerous studies demonstrated that an outstanding flame-retardant performance can be achieved by combining the flame-retardant additives with inorganic flame-retardant fillers, thereby decreasing the proportion of combustible polymers present [25,26,27]. Moreover, while the addition of a single filler is often less efficient, and does not meet the requisite flammability standards, studies suggest that the combination of multiple mineral fillers can greatly facilitate the flame retarding performance [28,29]. Presently, talc and calcium carbonate (CaCO_3_) are widely established as flame retardant fillers due to their affordability and thermal stability [30,31]. However, gypsum has attracted particular attention due to its environmental friendliness, cost-effectiveness, thermal stability, and excellent fire resistance [32,33,34,35,36]. Pure gypsum, also known as calcium sulfate dihydrate (CaSO_4_·2H_2_O), occurs naturally in crystal form with two water molecules in the crystalline structure. When the gypsum is exposed to heat, these water molecules are gradually released, thereby decreasing the temperature of the polymer matrix and reducing the oxygen concentration. Hence, the dehydrated calcium sulphate formed during combustion of the composite material settles onto the surface to form a protective layer of noncombustible material, thereby greatly contributing to the formation of a fire-resistant barrier against the transfer of heat and gas [37]. Moreover, a polymer binder can be incorporated in the composite material in order to consolidate the flame-retardant ingredients. In this respect, the industrial process is presently focused on the development of water-based formulations due to environmental concerns [38]. Hence, ethylene vinyl acetate emulsion (EVA) is presently used as a binder in the preparation of water-based formulations due to its good adhesion capacity and low-cost.

The present work examined the influence of gypsum upon a novel hybrid flame retardant (HFR) that incorporates ammonium polyphosphate (APP), pentaerythritol (PER), decabromodiphenyl ethane (DBDPE), expandable graphite (EG), calcium carbonate (CaCO_3_), and talc, which is applied onto the expanded polystyrene (EPS) foam. The thermal performance and flame retardancy of the as-fabricated EPS foam were investigated via a thermogravimetric analysis (TGA) and the cone calorimetry test (CCT). The results indicate that the optimized gypsum-based HFR plays a key role in boosting the flame resistance properties of the EPS foam, with a total heat release (THR) of 8 MJ/m^2^, a peak heat release rate (PHRR) of 53.1 kW/m^2^, and a fire growth rate (FIGRA) of 1682.95 W/m^−2^s. In addition, scanning electron microscopy (SEM), and energy dispersive spectroscopy (EDS) were used to investigate the combustion behavior of the residual char. To the best of the authors’ knowledge, this is the first time that the coating of EPS foam with a gypsum-based HFR material has been reported for improved flame-retardant performance.

## 2. Materials and Methods

### 2.1. Materials

The expanded polystyrene (EPS) beads, ammonium polyphosphate (APP, purity > 98%), pentaerythritol (PER, purity 98%), calcium carbonate (CaCO_3_, purity > 98.5%), decabromodiphenyl ethane (DBDPE, purity 99%), talc (whiteness: 94.0 ± 1%, particle size: 11.0 ± 2 μm), and EVA emulsion (G3, solid content 56.5%,) were obtained from HDC Hyundai EP Co., (Seoul, Korea). The expandable graphite (EG, purity: 99%, size 270 μm) was purchased from Yuil Chemi Tech Co. Ltd., (Seoul, Korea). The gypsum (purity > 96%) was provided by Namhae Chemical Corporation, (Yeosu, Korea).

### 2.2. Preparation of the Gypsum-Based HFR Formulation

The gypsum-based HFR materials were prepared according to the parameters listed in Table 1. In brief, a fixed amount of binder 55 g; (APP:PER:DBDPE:CaCO_3_ = 15:5:5:5 by mass) was added to 40 g of EG and 0, 9, 12, or 15 g of gypsum in 95, 104, 110, or 114 mL of distilled water, and stirred at room temperature for 48 h to obtain the flame-retardant solutions labelled as HFR0, HFR9, HFR12, and HFR15, respectively.

### 2.3. Preparation of the Flame-Retardant EPS Foam

A simple mixing method was used for the preparation of the flame-retardant EPS foam. When performing the typical procedure, EPS microspheres (13 g) and a HFR sample were mixed in a 1:3 ratio, and the uniformly coated EPS spheres were then transferred into a cuboid mold and hot pressed at 90 °C for 6 h. The cured cuboid EPS foam with the dimensions of 100 × 100 × 50 mm^3^ was carefully removed from the mold and dried in an oven at 60 °C for 24 h. The EPS samples that were prepared using HFR0, HFR9, HFR12, and HFR15 were correspondingly labelled as EPS, EPS/HFR0 EPS/HFR9, EPS/HFR12, and EPS/HFR15.

### 2.4. Characterization

The surface morphologies of the various EPS/HFR samples and the char residues obtained after combustion were recorded using a scanning electron microscope (SEM; S-4700, Hitachi). Thermogravimetric analyses (TGA; TA Instruments SDTA 851E) of the EPS and EPS/HFR samples were performed at a heating rate of 10 °C/min from room temperature (RT) to 800 °C under a nitrogen atmosphere. The flame-retardant behavior was measured by applying a butane spray gun jet at a distance of 5 cm from the cuboid HFR/EPS sample for complete combustion and the combustion test was monitored by recording digital photographs. To assess the flammability behaviors of the samples in a real fire, their peak heat release rate (PHRR), total heat release (THR), and fire growth rate (FIGRA) were evaluated via the cone calorimetry test (CCT) using a standard cone calorimeter (Fire Testing Technology Limited, UK) according to the ISO5660 standard under an external heat flux of 50 kW/m^2^ for 600 s. The thermal conductivity coefficient of the neat EPS and flame-retardant EPS foam was measured with a thermal conductivity analyzer (Dow chemical, Yeosu-si, Korea Ltd.). The specimen dimensions were 200 × 200 × 20 mm^3^.

## 3. Results and Discussion

### 3.1. Thermogravimetric Analysis (TGA)

**Figure 1:**
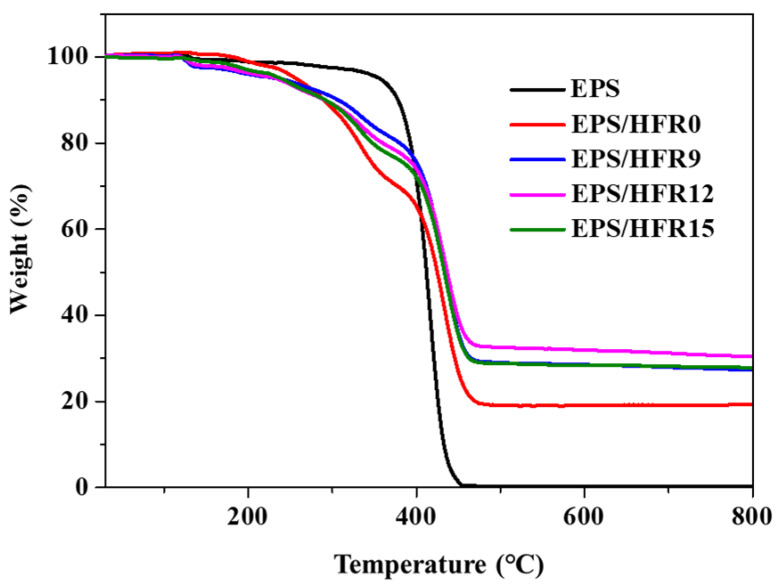
The TGA curves of the neat EPS and flame-retardant EPS samples obtained under a nitrogen atmosphere at a heating rate of 10 °C/min.

The thermal degradation behaviors of the various EPS samples are illustrated by the TGA curves in Figure 1. Here, the neat EPS clearly exhibited one-stage decomposition, with 100% weight loss taking place in the temperature range of 350 to 450 °C, so that no char residue remained after thermal decomposition [39]. By comparison, the hybrid EPS/HFR0 sample exhibited a significantly lower initial decomposition temperature, with a major weight loss between 180 and 460 °C due to the early decomposition of the EG and the increasing reaction between the flame-retardant additives, which led to a certain amount of residue char remaining at 800 °C. In detail, when the temperature rises above 180 °C, the EG begins to decompose and release sulfur dioxide [40,41], while the APP component of the binder begins to decompose to release water and ammonia; these decomposition products react to form polyphosphoric acid. Further, at temperatures between 200 and 300 °C, the polyphosphoric acid reacts with the hydroxyl group of the PER component of the binder to form a phosphate ester, which leads to the formation of char [42]. At 300–440 °C, however, the DBDPE component of the binder begins to decompose and release bromine radicals, which react to decrease the oxygen concentration and accelerate the gas phase, thereby boosting the flame retardancy [43,44,45]. Above 440 °C, an additional weight loss was observed due to the reaction of the talc and calcium carbonate components of the binder with the polyphosphate network [46,47,48]. With the addition of gypsum (CaSO_4_·2H_2_O), the initial decomposition temperature decreased slightly relative to that of EPS/HFR0 due to dehydration of the gypsum to form thermally stable calcium sulfate (CaSO_4_) [49]. This led to an increase in the final residual weight from 0% for the neat EPS and 19.30% for the EPS/HFR0 to 27.46, 27.80, and 30.52% for the EPS/HFR9, EPS/HFR15, and EPS/HFR12, respectively. These results suggest that the thermally stable CaSO_4_ interacts with HFR to form a thermally stable char layer structure, which acts as an effective barrier against heat and mass transfer during the combustion process, thereby enhancing the flame-retardant performance of the EPS/HFR foam.

### 3.2. Microstructural Study

**Figure 2:**
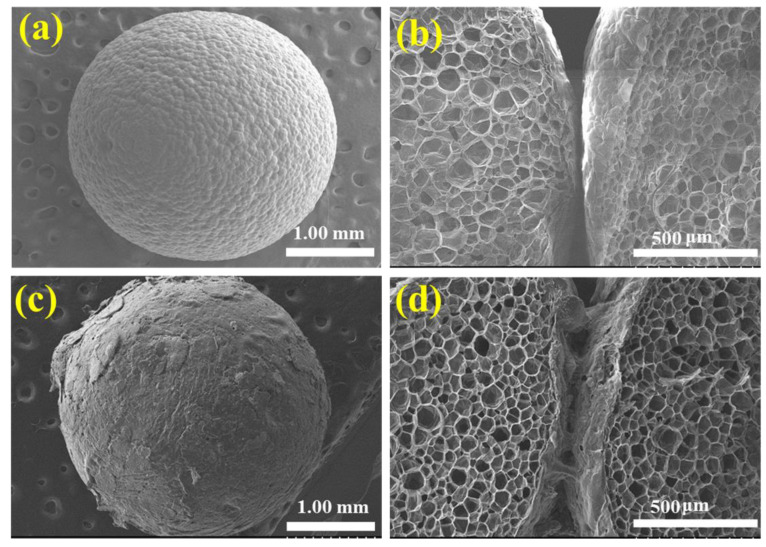
The SEM and cross-sectional SEM images of the neat EPS (**a**,**b**), and the EPS/HFR12 (**c**,**d**).

The surface morphologies of the EPS before and after the application of the flame-retardant coating are revealed by the SEM images in Figure 2. Here, the neat EPS exhibits a spherical shape with a very smooth surface morphology (Figure 2a). Further, the cross-sectional image in Figure 2b clearly shows the absence of any coating between the neat EPS beads. Due to its chemical composition, the EPS undergoes a radical chain reaction during combustion, thereby generating volatile products that can act as fuels for the production of toxic black smoke. By contrast, the SEM image of the EPS/HFR12 sample in Figure 2c confirms the successful coating of the EPS microsphere with the gypsum-based HFR materials, and the cross-sectional image in Figure 2d reveals the formation of the gypsum-based HFR coating between two adjacent EPS beads. During combustion, these flame-retardant coating materials can generate a compact char layer that can act as an effective fireproofing barrier, thereby improving the flame resistance performance of the EPS foam.

### 3.3. Combustion Behavior

**Figure 3:**
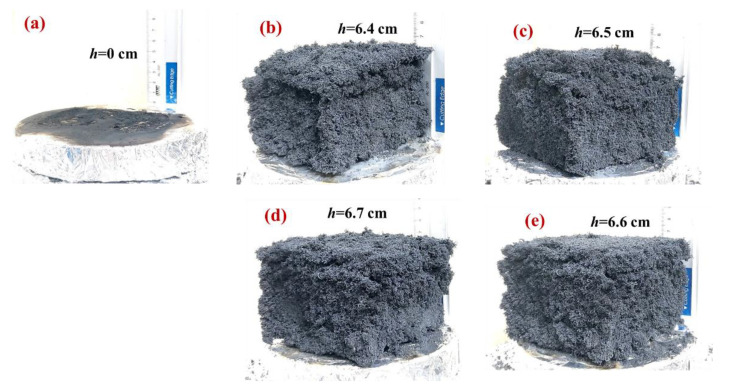
The digital photographs of the combusted samples: (**a**) the neat EPS, (**b**) the EPS/HFR0, (**c**) the EPS/HFR9, (**d**) the EPS/HFR12, and (**e**) the EPS/HFR15.

The photographic images of the neat EPS and the various flame-retardant EPS foams that were captured after the combustion test are presented in Figure 3. The neat EPS sample was observed to generate a smoky and sooty flame during the combustion process, and no residue was detected after combustion (Figure 3a). By contrast, the image in Figure 3b reveals the broken and expanded char layer and somewhat collapsed structure of the combusted EPS/HFR0. Moreover, although a similar char residue was observed for the flame-retardant EPS/HFR9 sample shown in Figure 3c, the structural integrity was better preserved than in the EPS/HFR0 sample. These results clearly demonstrate the improved flame-retardant performance of the coated EPS foam. Further, the increased gypsum content in the EPS/HFR12 sample was found to generate a dense and compact char foam without any cracking (Figure 3d). This can provide an even more effective thermal barrier, thus further enhancing the flame retardancy. However, the further increase in gypsum contents for the EPS/HFR15 sample led to the formation of voids in the char layer (Figure 3e). Here, the compactness and expansion ratio of the char layer were negatively impacted by the large amount of CaSO_4_, which impeded the diffusion of oxygen, heat, and combustible gases, thereby hindering the decomposition and volatilization of APP. Furthermore, the low integrity of this char layer ultimately reduces its flame-retarding behavior. These results demonstrate that a controlled content of gypsum plays key role in boosting the flame-retardant performance of the EPS foam, with the EPS/HFR12 sample providing the optimum effect.

### 3.4. Cone Calorimetry

**Figure 4:**
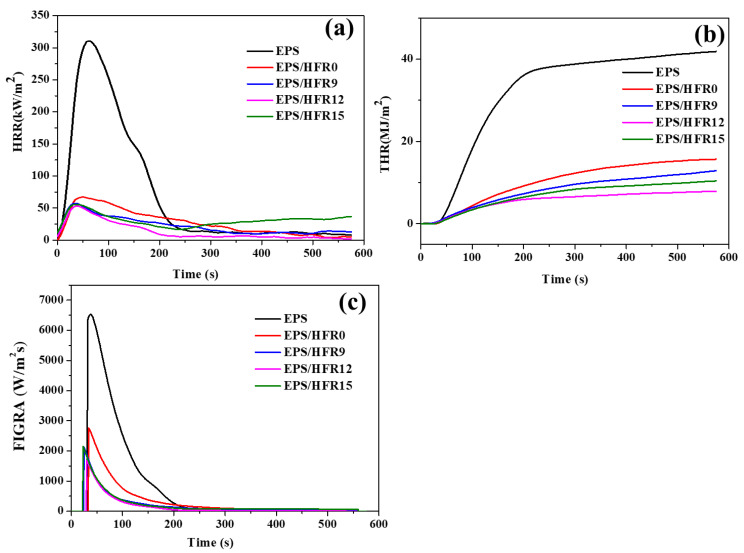
The cone calorimetry curves of the various EPS samples: (**a**) the PHRR curves; (**b**) the THR curves; and (**c**) the FIGRA curves.

The PHRR, THR, and FIGRA curves of the various samples are presented in Figure 4, and the numerical results are summarized in Table 2. Thus, the neat EPS ignited quickly, with high PHRR and THR values of 310.5 kW/m^2^ and 42.1 MJ/m^2^, respectively. After coating the hybrid flame-retardant onto the EPS surface, however, the EPS/HFR0 exhibited significantly reduced PHRR and THR values of 67.1 kW/m^2^ and 15.9 MJ/m^2^, respectively; these results confirm that the flame-retardant coating acts as a barrier layer during the combustion process. Furthermore, the addition of gypsum was seen to drastically reduce these values to 57.5 kW/m^2^ and 13.4 MJ/m^2^, respectively, for the EPS/HFR9 sample, and 53.1 kW/m^2^ and 8.0 MJ/m^2^, respectively, for the EPS/HFR12 sample. With the further increase in gypsum content, however, the PHRR and THR values increased slightly to 55.8 kW/m^2^ and 10.6 MJ/m^2^, respectively, for the EPS/HFR15 sample. These results further demonstrate that the gypsum-based flame-retardant coating has the potential to improve the flame retardancy of the EPS, with the EPS/HFR12 exhibiting by far the lowest PHRR and THR values.

The underlying mechanism for this improved flame-retarding behavior of the EPS foam in the presence of gypsum is as follows. During the combustion process, the gypsum absorbs the generated heat and releases water molecules to form the thermally stable calcium sulphate. During this endothermic process, a further increase in temperature is delayed until the gypsum is completely dehydrated. The resulting calcium sulphate then provides an effective barrier to further heat flow, thereby reducing the heat transfer during the remainder of the combustion process. The above results therefore demonstrate that gypsum plays a key role in boosting the flame retardancy of the HFR coating during the combustion process. However, the synergistic effect of the gypsum-based HFR relies on a moderate content of gypsum, with higher HRR and THR values observed when the gypsum content is increased in the EPS/HFR15 sample. The excess gypsum releases water to form excess thermally stable CaSO_4_ on the surface of the char layer, which not only hinders the diffusion of oxygen, heat, and flammable gases, but also hampers the decomposition and volatilization of the APP, thereby hindering the swelling process of the char layer and, thus, reducing the flame-retardant performance.

The burning characteristics of the materials are demonstrated by the FIGRA test results in Figure 4c and Table 2, where the lower FIGRA values of all the flame retardant-based EPS foams relative to the pristine EPS foam indicate the increased fire safety of the composite materials [50,51]. In detail, the EPS, EPS/HFR0, EPS/HFR9, EPS/HFR12, and EPS/HFR15 foams exhibit FIGRA values of 6530.8, 2764.1, 2119.0, 1682.9, and 2147.2 W/m^2^ s, respectively. Thus, the lowest FIGRA value was obtained with the flame-retardant EPS/HFR12 sample.

### 3.5. Char Residue Analysis

**Figure 5:**
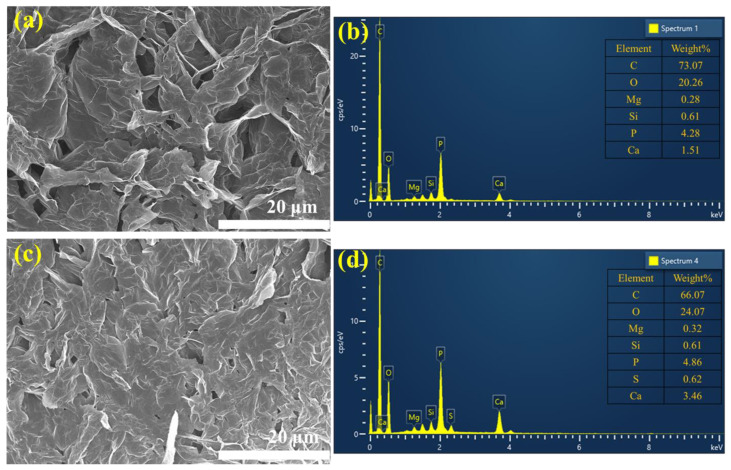
The SEM images (**a**,**c**) and corresponding EDS spectra (**b**,**d**) of the char residue on the EPS/HFR0 (**a**,**b**) and the EPS/HFR12 (**c**,**d**).

It is well known that the flame-retardant performance of the composite material depends on the compactness of the char layer that is clearly generated during the combustion process [52]. Hence, the role of the gypsum additive in the flame-retardant coating on the EPS foam was further elucidated by the SEM and EDS analysis of the char layer obtained in the CC test in the presence and absence of gypsum (Figure 5). Thus, the surface of the residual char layer on the EPS/HFR0 sample clearly exhibits some collapse structures and holes, which facilitate the release of large amounts of heat during the combustion process (Figure 5a). Further, the EDS analysis in Figure 5b indicates that the char residue on the EPS/HFR0 sample contains only C, O, Si, P, and Ca, with no S. In the presence of gypsum, however, the formation of a dense and compact residual char layer was detected on the EPS/HFR12 sample (Figure 5c), with severely limited voids compared to the EPS/HFR0 sample (Figure 5a). This more compact char would be beneficial for reducing heat transfer during the combustion process. Moreover, the EDX spectrum of the char residue on the EPS/HFR12 sample in Figure 5d reveals the presence of S, along with higher Ca and O contents than in the EPS/HFR0 (Figure 5b), clearly indicating the presence of the thermally stable CaSO_4_. Thus, the SEM-EDS results confirm the beneficial effects of the gypsum additive in enhancing the flame-retardant performance of the EPS foam not only via the endothermic dehydration process and formation of the insulating CaSO_4_ layer, as detailed above, but also via the expansion of the EG, which releases non-flammable gases and generates a worm-like char layer. Similarly, the APP content of the binder begins to decompose and release incombustible gases such as NH_3_ and H_2_O, thereby resulting in the formation of polyphosphoric acid, as detailed in Section 3.1. The esterification reaction between this phosphoric acid and the hydroxyl group of the PER in the binder then results in the formation of a char framework. In addition, the DBDPE content of the binder decomposes and releases bromine radicals, which accelerate the gas phase and suppress the spreading of the flame. Meanwhile, the talc and CaCO_3_ components react with the phosphoric network to form a silicon phosphate and calcium phosphate, thereby resulting in the formation of a thermally stable and dense, compact char, which further enhances the flame resistance of the EPS foam by helping to reduce the PHRR and THR during combustion. Thus, the as-fabricated gypsum-based HFR materials can effectively limit the combustion process of the EPS material.

### 3.6. Physical Properties

In order to use a material for thermal insulation application, the EPS foam must not only be able to fulfill the demand for flame-retardant performance, but it should also possess essential physical properties, such as density and thermal conductivity. The influence of gypsum with hybrid flame retardant materials on the density and thermal conductivity of EPS foams was tested, and the results are listed in Table 3. In comparison to neat EPS foam, the density of flame-retardant-based EPS foam increased. The neat EPS foam exhibited a density of 26 kg/m^3^, which increased to 68 kg/m^3^ for EPS/HFR0. We observe that with the incorporation of gypsum, the density of flame-retardant EPS improved to up to 71 kg/m^3^ (for EPS/HFR9), 72 kg/m^3^ (for EPS/HFR12), and 74 kg/m^3^ (for EPS/HFR15). This increased density may be caused by the uniform adhesion of the flame-retardant coatings on the surface of the EPS beads, which may be attributed to the increase in gypsum content in hybrid flame retardant systems.

The thermal conductivity is a vital index for measuring the thermal insulation performance of EPS foam. This signifies its suitability in thermal insulating applications. Our results indicate that neat EPS exhibits a very low thermal conductivity 0.028 W/m.K. However, we observed an enhanced thermal conductivity of up to 0.038 W/m.K for the EPS/HFR0 system. It was also found that the thermal conductivity of flame-retardant EPS with and without gypsum contents remained unchanged and was observed as identical to the 0.038 W/m.K value. It was determined that the thermal conductivity of flame-resistant EPS foam may be significantly enhanced with acceptable thermal insulation properties.

## 4. Conclusions

A gypsum-based hybrid flame retardant (HFR) system was prepared herein in order to boost the flame-retardant performance of expanded polystyrene (EPS)-based foam materials. The morphological analysis confirmed that the gypsum-based HFR layer was uniformly coated on the EPS beads. In addition, thermogravimetric analysis (TGA) showed that the gypsum significantly enhanced the final residual weight at 800 °C. Importantly, the cone calorimetry test (CCT) results showed that the addition of an optimum amount (12 g per 55 g of binder) of gypsum effectively reduced the peak heat release rate (PHRR), total heat release (THR), and fire growth rate (FIGRA) values to 53.1 kW/m^2^, 8 MJ/m^2^ and 1682.95 W/m^−2^ s, respectively. In addition, the char residue analysis demonstrated that the incorporation of gypsum provides a thermally stable and compact char layer, thereby boosting the flame-retardant properties of the EPS foam. However, an excessive amount of gypsum (15 g per 55 g of binder) was found to restrict the formation of the hybrid char products and destroy the swelling behavior of the charred layer, thereby compromising the flame-retardant performance of the HFR. The authors believe that the addition of the optimum amount of gypsum (12 g per 55 g of binder) provides HFR with promising flame-retardance and satisfies the fire-safety standards.

## Figures and Tables

**Table 1:** The preparative parameters of the gypsum-based HFR formulations.

Sample	Binder ^a^ (g)	Gypsum (g)	EG (g)	Water (mL)
HFR0	55	0	40	95
HFR9	55	9	40	104
HFR12	55	12	40	110
HFR15	55	15	40	114

^a^ Hybrid flame retardant additive composition was used as APP:PER:DBDPE:CaCO_3_ =15:5:5:5 by mass.

**Table 2:** The cone calorimeter test results for the various EPS foam samples.

Sample	PHRR (kW/m^2^)	THR (MJ/m^2^)	FIGRA (W/m^2^·s)
EPS	310.5	42.1	6530.8
EPS/HFR0	67.1	15.9	2764.1
EPS/HFR9	57.5	13.4	2119.0
EPS/HFR12	53.1	8.0	1682.9
EPS/HFR15	55.8	10.6	2147.2

**Table 3:** Physical properties of the neat EPS and flame-retardant-based EPS foam.

Sample	Density (kg/m^3^)	Thermal Conductivity (W/m.K)
EPS	26	0.028
EPS/HFR0	68	0.038
EPS/HFR9	71	0.038
EPS/HFR12	72	0.038
EPS/HFR15	74	0.038

## Data Availability

All data has been provided in within this manuscript.
